# *Notice to Readers:* New Web Location for Annual and Weekly NNDSS Data

**DOI:** 10.15585/mmwr.mm6638a6

**Published:** 2017-09-29

To improve the usability, availability, quality, and timeliness of surveillance data (*1*) as part of the CDC Surveillance Strategy, CDC will provide users with a convenient way to access notifiable infectious and noninfectious disease data through the National Notifiable Diseases Surveillance System (NNDSS) website.

CDC has redesigned the data and statistics section of the NNDSS website to be a one-stop shop for users to find both detailed information about notifiable disease data and links to the annual and weekly data. Although these data will no longer be published in their current format in *MMWR*, users can easily access the information through the NNDSS website. To ease the transition, *MMWR* also will link users from its website to the new location on the NNDSS website.

## Annual Reporting

CDC expects to transition the reporting of NNDSS annual data in November 2017. The redesigned NNDSS Data and Statistics webpage at https://wwwn.cdc.gov/nndss/data-and-statistics.html will contain links to infectious disease data tables that are available in HTML, text, and PDF formats and hosted on the CDC WONDER (*2*) platform. The webpage also will provide links to noninfectious condition and disease outbreak surveillance reports published by CDC programs and hosted on CDC WONDER. In addition, the webpage will provide the following resources: 1) documentation for NNDSS infectious diseases and noninfectious conditions and disease outbreaks, including how the data are collected, reported, and finalized; 2) publication criteria; 3) notes about interpreting data; and 4) the list of notifiable conditions by year.

## Weekly Reporting

CDC expects to transition the reporting of NNDSS weekly data in January 2018 and will provide more information later this year.

Consolidating the notifiable disease data on the NNDSS website is part of the NNDSS Modernization Initiative (NMI) strategy to streamline NNDSS and access to data for users; NMI is a component of the CDC Surveillance Strategy. This consolidation of information also is in response to the recommendations of a workgroup consisting of representatives from the CDC Excellence in Science Committee, the Surveillance Science Advisory Group, and *MMWR*, to make more data available online and to allow *MMWR* to focus on publishing scientific and actionable surveillance reports.
